# Carrot Powder and Jaggery Supplemented Germinated Oat, Teff Based Complementary Infant Food: Formulation, Optimization, and Characterizations

**DOI:** 10.1002/fsn3.70264

**Published:** 2025-08-06

**Authors:** Ramesh Duraisamy, Bahiru Bekele, Belay Haile, Eyob Mulugeta, Tanje Mada

**Affiliations:** ^1^ Department of Chemistry (Industrial Chemistry Division) College of Natural and Computational Sciences, Arba Minch University Arba Minch Ethiopia

**Keywords:** anti‐nutrients, carrot, complementary food, germinated cereals, jaggery, teff

## Abstract

The complementary food value has increased over the last decades and is expected to continue rising. The study focused on developing and evaluating a nutritious composite complementary Infant food alternative with lower antinutrients than the commercial one. The current study was undertaken by formulating (using Minitab software, v.19.1, USA) germinated Oats and Teff supplemented with Carrot powder and Jaggery. The proximate composition of formulated complementary Infant food was analyzed, and the reported values ranged from 7.13 ± 0.03 to 13.31 ± 0.01 g (moisture), 2.76 ± 0.03 to 3.35 ± 0.02 g (ash), 7.83 ± 0.02 to 15.97 ± 0.03 g (protein), 2.68 ± 0.02 to 4.27 ± 0.02 g (fat), 2.67 ± 0.02 to 12.11 ± 0.02 g (fiber), 54.87 ± 0.02 to 69.34 ± 0.03 g (carbohydrates), and energy: 301.62 ± 0.02 to 360.47 ± 0.03 kcal/100 g. The studied results reported a better food composition: 59.5 g of Oat‐Teff (1:1 mixture), 30.5 g of Carrot powder, and 10 g of Jaggery supported with a maximum desirability of 0.9736. The sensory attributes (using a seven‐point hedonic scale) of formulated best‐optimized complementary food compositions were 6.24 ± 0.49 (taste), 6.39 ± 0.13 (flavor), 6.51 ± 0.04 (color), 5.84 ± 0.14 (texture), 6.47 ± 1.24 (appearance), 5.87 ± 0.73 (mouthfeel), and 6.55 ± 0.58 (overall acceptability). Thus, the studied results conclude that the formulated optimized composite food is promising, unique, and value‐added to the coarse cereals (Oat and Teff), Carrot, and Jaggery under the complementary composite food category of Infant food.

## Graphical abstract

The current study was undertaken by formulating (using Design‐Expert software, v. 13.1, USA) germinated Oats and Teff supplemented with Carrot powder and Jaggery. The studied results reported a better food composition: 59.5 g of oat‐teff (1:1) mixture, 30.5 g of carrot powder, and 10 g of jaggery supported with a maximum desirability of 0.9736. The better‐optimized complementary food had a good nutritional profile and lower antinutrients compared to the World Health Organization's standard recommended daily intake level.

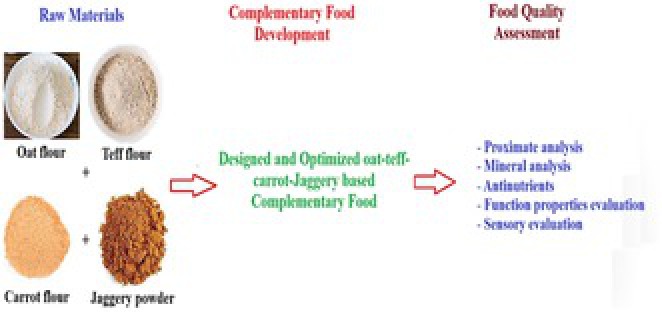

## 1 Introduction

Complementary foods are solids, semisolids, and liquid foods other than human milk or Infant formula provided to an Infant or young child to afford nutrients and energy (Dewey and Brown 2003; Wakil and Ola 2018). Food composites blend various food groups' characteristics, enabling products with improved or new functional and nutritional properties. Cereal‐based foods and vegetables, including complementary food products, play a vital role in the human diet, contributing significantly to the average intake of each food component (Alexandratos and Jella 2012). There is a growing consumer interest in healthier and more wholesome nutrition (Sirό et al. 2008). Consumers perceive these foods as achieving satisfaction, preventing diseases, and maintaining control over their health (Sirό et al. 2008; Corbo et al. 2014). Additionally, complementary foods should ideally be a complete source of bioactive components, which can be achieved through food processing methods such as germination (Gibson and Williams 2000).

Germination involves natural physiological, morphological, and chemical changes that alter the functional properties of cereals (Ahmed et al. 2006). During this process, partially hydrolyzed starch, protein, and fat degradation enhance these nutrients' digestibility. It also improves the functional attributes of legumes and seeds, reduces their water absorption capacity, and increases malted millet's oil/fat absorption capacity (Agrawal et al. 2013).

Iron is the most widespread micronutrient deficiency worldwide, impacting over two billion individuals (Rohner et al. 2010). This deficiency often results from insufficient intake of bioavailable iron or increased physiological demands, particularly during pregnancy and early childhood (Pasricha et al. 2013). According to the World Health Organization (WHO), fortifying foods with iron is considered the most effective strategy to prevent iron deficiency. Supporting this, Bouhouch et al. (2016) demonstrated that consuming iron‐fortified foods significantly enhances iron status.

A systematic review of effectiveness trials found that consuming iron‐fortified foods enhances iron availability and lowers the risk of anemia (Assuncao et al. 2007; Glinz et al. 2017). In regions prone to vitamin A deficiency, improving vitamin A levels through dietary intake of provitamin A carotenoids may be more beneficial than periodic supplementation with preformed vitamin A (Tang et al. 2005). Carrots are well known for their high β‐carotene content, providing 210% of the average adult's daily vitamin A requirement (Tang et al. 2005). Jaggery, made by concentrating sugarcane juice through continuous heating, offers better nutritional value than table sugar and is considered a superior alternative sweetener in the food industry (Dilip et al. 2017). Commercially available complementary foods, often imported, are processed into solid or liquid (gruel) forms and typically contain corn flour and milk powder as main ingredients. These commercial foods usually include sucrose for sweetening (Fardet 2015).

The present research investigated the nutritional, antinutritional, and functional properties of germinated cereal‐based vegetable‐added complementary food products and intends to use Jaggery as an alternative to sucrose in sprouted Oat‐Teff‐based Carrot powder fortified complementary food preparation.

## 2 Materials and Methods

### 2.1 Sample Collection

The samples of the chosen cereals (Oat, *Avena sativa* , and Teff, *Eragrostis tef* ) were procured from the Arba Minch, South Ethiopia, local market. Sugarcane was collected from the Mirab Abaya sugarcane cultivation farms, which are located in South Ethiopia. The collected samples were transported to the laboratory and preserved appropriately for further processing.

### 2.2 Cereal Germination

Germination of cereals was processed by employing the standard procedure described earlier (Elena et al. 2017; Papastylianou et al. 2019) with slight modifications.

### 2.3 Carrot Powder Preparation

Matured and fresh Carrots ( *Daucus carota* ) were collected from the cultivation land at Mirab Abaya in South Ethiopia. The samples of Carrots were pretreated by washing them with demineralized water and blanching them in boiling water for 10 min. Then, the Carrot was smashed and introduced in an oven to dry (80°C). Then, the dried slices of Carrot were ground using a laboratory blender and sieves using 0.5 mm mesh (Debasis Roy 2018; Jain and Singh 2021). The prepared Carrot powder (as a blended foodstuff) was stored in the refrigerator to analyze and prepare composite food.

### 2.4 Preparation of Jaggery

Prepared the sugarcane Jaggery under three‐step processes: juice extraction, clarification, and concentrating by evaporation (Dilip et al. 2017; Aynalem and Ramesh 2022). The obtained moist granular Jaggery, which was sieved through 3 mm, was then dried in the shade for 2–3 h and sun‐dried for 30 min to reduce the moisture content to lower than 2% (Aynalem and Ramesh 2022). Then, the obtained low‐moist granular Jaggery was packed and preserved in moisture‐free polyethene bags for further analysis. It was used as a blending foodstuff to prepare composite food.

### 2.5 Preparation and Optimization of Complementary Food

The optimization was carried out by keeping the three interchangeable ratios of composite flour of germinated Oat, germinated Teff (1:1 ratio), Carrot powder, and Jaggery ranging from 50–65 g, 24–35 g, and 10–15 g, respectively (sample code: OS_1_–OS_13_). The total composition of composite flour was taken as 100%. The complementary food was prepared (as shown in Table 1) using a simplex mixture design using Minitab software version 19.1.

**TABLE 1:** Proximate composition of foodstuffs (raw materials) of 100 g.

Raw materials	Proximate composition for raw materials (g/100 g)						Energy (kcal.)
	Moisture	Ash	Fiber	Fat	Protein	A. CHO	
Teff	6.83 ± 0.02^i^	2.87 ± 0.02^gh^	7.22 ± 0.02^g^	3.62 ± 0.02^de^	9.72 ± 0.02^k^	69.74 ± 0.02^b^	350.66 ± 0.02^d^
Oat	7.23 ± 0.15^hi^	1.41 ± 0.03^k^	6.72 ± 0.02^h^	4.62 ± 0.03^a^	14.06 ± 0.12^d^	66.17 ± 0.3^f^	362.10 ± 0.02^a^
Carrot powder	16.47 ± 0.02^a^	6.65 ± 0.02^a^	10.25 ± 0.02^c^	2.96 ± 0.02^fgh^	4.35 ± 0.02^n^	64.94 ± 0.02^h^	303.86 ± 0.03^p^
Jaggery	12.35 ± 1.76^bc^	2.38 ± 0.02^j^	0.04 ± 0.01^n^	2.20 ± 0.03^i^	6.37 ± 0.02^m^	72.57 ± 0.02^a^	335.40 ± 0.02^k^

*Note:* Mean do not share the same superscripts in column are significantly different (*p* < 0.05). A: 1:1 oat/teff mixture (g); B: amount of carrot powder (g); C: amount of jaggery (g).

Abbreviation: A. CHO, available carbohydrates.

### 2.6 Evaluation of Raw Materials and Prepared Complementary Foods

#### 2.6.1 Proximate Composition and Mineral Analysis

Proximate components of the studied foodstuffs (raw materials) and formulated food samples were analyzed by adopted AOAC method numbers: moisture, 925.10; ash content, 923.03; crude fat, 920.39; protein, 962.09; available carbohydrates, 2020.07; and for minerals (Ca, K, Na, Fe, and Zn), 984.27, which are described in the AOAC manual (AOAC 2023).

#### 2.6.2 Measurement of Gross Energy

Determined the gross energy of raw materials and prepared complementary food according to the AOAC (2023):

### 2.7 Determination of Antinutritional Components

#### 2.7.1 Phytate Content

The amount of phytate was quantified by employing the method described by Tabitha et al. (2020). The sample (5 g) weighed accurately and then introduced into a conical flask containing 100 mL of 2% HCl; after that, it was soaked for 5 h. Then, it was filtered, 25 mL of filtrate was taken, and 0.3% ammonium thiocyanate (5 mL) was added. Titrating the mixture with std. FeCl_3_ solution appeared to be a golden color after 5 min. The phytate content (Tabita et al. Tabitha et al. 2020) as follows:

(1) $$\text{Phytic acid}=\text{Titer value}\;\left(0.00195\right)\;1.19\times 100$$

Where 0.00195 is the weight of FeCl_3_.

#### 2.7.2 Bioavailability of Minerals Concerning Phytate

Bioavailability refers to the proportion of an ingested nutrient in food that is absorbed and utilized through normal metabolic pathways. It is influenced by both host and diet‐related factors (Gibson et al. 2010). The molar ratio of anti‐nutrients/minerals was used to predict the mineral's bioavailability. The molar ratio of phytate values to studied minerals (Fe, Ca, Zn, and Na) was determined by dividing the weight of phytate and minerals by their atomic weight according to the earlier study (Kumari et al. 2014).

#### 2.7.3 Estimation of Oxalate

The samples (4 mL) were suspended for each treatment in 190 mL of distilled water; it was taken to a 250 mL Erlenmeyer flask, 6 M HCl (10 mL), and the suspension was digested at 100°C for 1 h. The mixture was allowed to cool, and the solution was made up to the mark (250 mL) using distilled water and filtered into another flask.

Two 125 mL portions of the filtrate were measured and transferred into beakers. To each portion, 3–4 drops of methyl red indicator were added, followed by the dropwise addition of concentrated ammonium hydroxide until the solution's color changed from salmon pink to a pale yellow. The pH was recorded at this stage. Each solution was then heated to 90 °C, allowed to cool, and filtered to eliminate the precipitate containing ferrous ions. The resulting filtrate was reheated to 90 °C, after which 10 mL of 5% calcium chloride solution was added while stirring continuously.

Then, the sample was cooled and left overnight at 5°C and centrifuged in the solution at 2500 rpm for 5 min. The supernatant was decanted, and the precipitate was dissolved in 10 mL of 20% (v/v) sulfuric acid solution. The total filtrate was obtained from the digestion of 4 mL of the complementary food sample, which was diluted to 300 mL. Aliquots (125 mL) of the filtrate were heated till boiling. Titration was carried out against 0.079 M standardized potassium permanganate solutions until it got a faint pink color, which persisted for 30 s. Calculated oxalate content according to AOAC method, 952.03 (AOAC 2023) as:

(2) $$\text{Oxalate content}\;\left(\%\right)=\frac{T\;x\;\left(\mathrm{Vme}\right)\;x\;\left(\mathrm{Df}\right)\;x\;10^{5}}{\mathrm{Me}\;x\;\mathrm{Mf}}$$

Where T is the titer value of KMnO_4_ (mg/100 g); Vme is the volume‐mass equivalent in which 1 cm^3^ of 0.079 M KMnO_4_ = 0.00225 g anhydrous oxalic acid; D_f_ is the dilution factor; M_e_ is the molar equivalent of KMnO_4_ in oxalate–KMnO_4_ redox reaction; M_s_ is the sample mass.

### 2.8 Evaluation of Functional Properties of Complementary Food

#### 2.8.1 Swelling Capacity

Swelling capacity (SC) and protein solubility index (PSI) were assessed following the procedure outlined by Shad et al. (2011). A uniform dispersion was prepared by mixing 0.2 g of flour with 10 mL of distilled water. The resulting slurry was then heated at 60 °C using a temperature‐controlled water bath. After heating, the mixture was allowed to cool to room temperature and centrifuged at 2200 rpm for 15 min. After centrifugation and retaining water, the obtained residue was reweighed. Calculate the SC by using the following equation:

(3) $$\mathrm{SC}=\frac{\mathrm{Wrw}}{\mathrm{Ws}}\times 100$$

Where Ws is the sample weight; Wrw is the sample weight with retained water.

The supernatant from the swelling capacity producer was heated at 100°C and the obtained residue was weighed, and the protein solubility index (PSI) of complementary food in water was calculated as follows:

(4) $$\mathrm{PSI}\;\left(\%\right)=\frac{\mathrm{Wrc}}{\mathrm{Ws}}\times 100$$

Ws is the sample weight; W_rc_ is the residue weight after centrifugation and evaporation.

#### 2.8.2 Bulk Density

Chinma et al. (2008) described the procedure and determined flour's bulk density. Accordingly, the flour (10 g) in a preweighed (W_1_) measuring cylinder, the weight of the cylinder with the sample (W_2_), and the volume of the flour (V_1_) were noticed. Then, the bulk density of the samples was calculated (in g/cc) as:

(5) $$\text{Bulk density}\;\left(/\right)=\frac{w2-w1}{v1}$$

W_1_—weight of the cylinder; W_2_—weight of the cylinder with the sample; V_1_—volume of the flour.

#### 2.8.3 Water Absorption Capacity

Measured samples' water absorption capacity (WAC) by centrifugation (Khan and Saini 2016). Distilled water (30 mL) was added to the sample (5 g) of flour containing a preweighed centrifuge tube and stirred six times at 10‐min intervals. Then, the centrifuged mixture was run at 3000 rpm for 30 min; then, it was decanted, and the clear supernatant was discarded. The residue was dried at 50°C for 30 min, and the adhered drops of water were removed and weighed. The water retained in the sample was recorded as weight gain and taken as water absorbed. Finally, the water absorption capacity is expressed as the weight of water bound by 100 g of dried flour:

(6) $$\mathrm{WAC}\;\left(g/g\right)=\frac{\text{Weight of sample}+\text{tube after heating}-\text{Sample weight}\;}{\text{sample weight}}$$

#### 2.8.4 Oil Absorption Capacity

Oil absorption capacity (OAC) was performed (Karklina et al. 2012) by the addition of refined sunflower oil (10 mL, density of 0.92 g/L) into the sample (2 g) containing a 30 mL centrifuge tube. Then, the resulting suspension was stirred using a magnetic stirrer for 5 min and centrifuged (at 3600 rpm) for 30 min; then, the suspension was measured using 10 mL of the graduated cylinder. The oil absorption capacity was determined by measuring the difference between the initial volume of oil added to the sample and the volume of the supernatant obtained after processing. The analysis was performed in triplicate, and the results were expressed as grams of oil absorbed per gram of the sample. The oil absorption capacity was then calculated accordingly as follows:

(7) $$\mathrm{OAC}\;\left(g/g\right)=\frac{\left[\left(\mathrm{Wt}\;\text{of the tube with sample after drying}\right)-\mathrm{Wt}\;\text{of}\;a\;\text{tube}\right)]-\text{Sample weight}}{\text{Sample weight}}$$

### 2.9 Determination of β—Carotene

The amount of β—carotene was estimated using the method stated by Felistus et al. (2017). Calculated the concentration (in mg/L^−1^) using Beer–Lambert's law from the absorbance by employing the equation as follows:

(8) $$C=\frac{10^{3}x\;\mathrm{MA}}{\mathrm{L\epsilon}}$$

Where: C is the concentration of β—carotene; M is the molecular weight of β‐carotene (536.8 g/mol); L is the path length (equal to 1 cm); ϵ is the molar extinction coefficient for β—carotene in petroleum ether (138,900 L/mol/cm).

### 2.10 Sensory Evaluation of Complementary Foods

#### 2.10.1 Preparation of Gruel

Porridge of optimized composite flours and control was prepared in water and boiled for 5 min with constant stirring (Catherine et al. 2015). They were then kept in a water bath and maintained at 54°C–56°C, a range of recommended consumption temperature for porridges for young children (Catherine et al. 2015). Then, prepared porridges were kept ready to measure the sensory attributes.

#### 2.10.2 Sensory Evaluation

Sensory evaluation was carried out with 35 semitrained panelists between the ages of 25 and 40. Questionnaires were distributed to assess the sensory attributes of the samples based on specific criteria. The parameters evaluated included color, taste, flavor, texture, appearance, mouthfeel, and overall acceptability. A 7‐point hedonic scale was used for scoring, where seven indicated “like extremely,” 6 “like very much,” 5 “like slightly,” 4 “neither like nor dislike,” 3 “dislike slightly,” 2 “dislike moderately,” and 1 “dislike very much” (Ji‐Woo‐Yoon et al. 2017).

### 2.11 Statistical Analysis

The collected data were statistically analyzed using analysis of variance (ANOVA) with Minitab software (version 19.1, USA). *p*‐values were employed to determine statistically significant factors, and mean comparisons were performed using the Tukey test. Differences between means were considered significant at a 95% confidence level (*p* ≤ 0.05).

## 3 Results and Discussion

### 3.1 Proximate Composition and Gross Energy of Raw Materials

All raw materials' proximate components (ash, moisture, fat, fiber, protein, and carbohydrates) were analyzed, and the obtained results are given in Table 1.

#### 3.1.1 Moisture

Available moisture in food samples is vital because it determines the storage conditions, such as shelf life and food safety. As seen in the experimental results (Table 1), the raw materials moisture content is 6.83 ± 0.02 g/100 g (Teff), 7.23 ± 0.15 g/100 g (Oat), 16.47 ± 0.02 g/100 g (Carrot powder), and 12.35 ± 1.76 g/100 g (Jaggery). The moisture content of the currently studied raw materials ranged from 6.83 ± 0.02 g/100 g to 16.47 ± 0.02 g/100 g.

#### 3.1.2 Ash

The ash content in the raw material ranges from 1.41 ± 0.03 g/100 g to 6.65 ± 0.02 g/100 g, as shown in Table 1. From the raw materials, Carrot powder has significantly higher ash content (6.65 ± 0.02 g/100 g) than other ingredients since Carrot contains a variety of minerals, and the results were found to be aligned with the results reported in the earlier study (Roshana and Mahendran 2019). Oat and Jaggery (used as a sweetener) had lower ash content.

#### 3.1.3 Fiber

Studied raw materials were reported as 7.22 ± 0.02 g (in Teff), 6.72 ± 0.02 g (Oat), and 10.25 ± 0.02 g (Carrot powder) of fiber content. However, Jaggery has a lower fiber content (0.04 ± 0.01/100 g), which indicates that it is prepared from well‐clarified cane juice. The fiber content of the prepared complementary food ranged from 2.67 ± 0.02 to 12.11 ± 0.02 g/100 g (shown in Table 1). According to Praveen Kumar et al. (2017), Oat is an excellent source of dietary fiber (β‐glucan); this is supported by formulating Oat‐based complementary foods to achieve the nutrient requirement of the consumers (Infants and any age holders).

#### 3.1.4 Fat

Among the presently studied foodstuffs, Oat powder reported a significantly higher fat content (44.62 ± 0.03 g). Since Oat is rich in mono‐unsaturated fats, it is an excellent fat supplement for formulating complementary Infant foods.

#### 3.1.5 Protein

The raw materials reported the protein content to be 9.72 ± 0.02 g (Teff), 14.06 ± 0.12 g (Oat), 4.35 ± 0.02 g (Carrot powder), and 6.37 ± 0.02 g of Jaggery. Furthermore, Teff and Oat used in the present study were germinated cereals; this helps improve the protein content due to enzymatic activities like proteinase enzymes, which microorganisms might produce and reduce antinutritional factors.

#### 3.1.6 Available Carbohydrates

The estimated carbohydrate content of raw materials is shown in Table 1 as 69.74 ± 0.02 g, 66.17 ± 0.3 g, 64.94 ± 0.02 g, and 72.57 ± 0.02 g for Teff, Oat, Carrot powder, and Jaggery, respectively. Jaggery has more carbohydrates due to the processing of cane juice, which mainly contains sucrose with a small amount of other sweetening saccharides.

### 3.2 Optimization of Complementary Food Formulation Using Food Constituents

#### 3.2.1 Using Proximate Components and Gross Energy

Proximate components (moisture, ash, fat, fiber, protein, and carbohydrates) were used for the formulated composite, which was analyzed for optimization. The obtained results are displayed in Table 2.

**TABLE 2:** Proximate composition of prepared composite complementary foods.

Food mixture ratio (g)				Proximate composition of formulations (g/100 g)						Energy (kcal.)
Code	A	B	C	Moisture	Ash	Fiber	Fat	Protein	A. CHO	
OS_1_	65.6	24.2	10.2	9.66 ± 0.04^defg^	2.76 ± 0.03^i^	5.65 ± 0.03^k^	2.68 ± 0.02^h^	9.89 ± 0.02^k^	69.34 ± 0.03^c^	341.06 ± 0.02^f^
OS_2_	63.0	26.3	10.7	10.35 ± 0.02^def^	3 ± 0.02^ef^	6.5 ± 0.02^i^	3.36 ± 0.01^ef^	11.82 ± 0.02^h^	64.97 ± 0.03^h^	337.27 ± 0.01^j^
OS_3_	63.7	25.0	11.3	9.86 ± 0.02^defg^	2.87 ± 0.02^gh^	2.67 ± 0.02^m^	4.05 ± 0.6^bced^	11.31 ± 0.02^i^	68.96 ± 0.02^d^	360.47 ± 0.03^b^
OS_4_	62.2	27.8	10.0	11.06 ± 0.02^cd^	3.04 ± 0.02^e^	8.54 ± 0.02^d^	4.27 ± 0.02^ab^	13.83 ± 0.11^e^	59.33 ± 0.02^o^	330.64 ± 0.02^m^
OS_5_	58.1	30.0	11.9	10.42 ± 0.02^def^	2.91 ± 0.02^fg^	7.36 ± 0.02^f^	3.15 ± 0.02^fg^	13.37 ± 0.01^f^	62.81 ± 0.02^l^	333.07 ± 0.01^l^
OS_6_	61.9	25.7	12.4	11.13 ± 1.75^cd^	2.78 ± 0.02^hi^	7.23 ± 0.03^g^	3.82 ± 0.02^cd^	12.81 ± 0.15^g^	60.23 ± 0.02^n^	326.53 ± 0.03^n^
OS_7_	55.8	31.2	13.0	7.13 ± 0.03^hi^	3.40 ± 0.02^b^	8.24 ± 0.02^e^	2.85 ± 0.02^gh^	15.97 ± 0.03^a^	62.39 ± 0.03^m^	339.21 ± 0.03^h^
OS_8_	54.1	32.5	13.4	8.23 ± 0.03^ghi^	3.23 ± 0.02^d^	6.35 ± 0.03^j^	4.16 ± 0.03^bc^	14.56 ± 0.02^c^	63.57 ± 0.02^j^	349.70 ± 0.02^e^
OS_9_	51.2	35.0	13.8	7.81 ± 0.03^hi^	3.35 ± 0.02^bc^	5.26 ± 0.01^l^	3.72 ± 0.02^cde^	15.42 ± 0.03^b^	64.43 ± 0.03^i^	352.66 ± 0.02^c^
OS_10_	52.4	33.1	14.5	8.77 ± 0.02^efgh^	3.28 ± 002^cd^	6.53 ± 0.02^i^	2.78 ± 0.03^gh^	13.45 ± 0.02^f^	65.24 ± 0.02^g^	340.15 ± 0.02^g^
OS_11_	50.6	34.2	15.2	8.62 ± 0.02^fghi^	3.19 ± 0.02^d^	11.23 ± 0.02^b^	3.68 ± 0.01^de^	10.34 ± 0.02^j^	62.95 ± 0.02^k^	326.21 ± 0.03^o^
OS_12_	57.4	26.6	16.0	13.31 ± 0.01^b^	2.85 ± 0.12^ghi^	12.11 ± 0.02^a^	2.98 ± 0.03^fgh^	13.85 ± 0.02^e^	54.87 ± 0.02^p^	301.62 ± 0.02^q^
OS_13_	58.7	28.5	12.8	10.54 ± 0.01^cde^	3.00 ± 0.02^ef^	6.39 ± 0.02^j^	3.65 ± 0.02^de^	7.83 ± 0.02^l^	68.58 ± 0.01^e^	338.56 ± 0.03^i^

*Note:* Mean do not share the same superscripts in column are significantly different (*p* < 0.05). A: 1:1 oat/teff mixture (g); B: amount of carrot powder (g); C: amount of jaggery (g).

Abbreviation: A. CHO, available carbohydrates.

##### 3.2.1.1 Moisture Content

The moisture content of the presently studied complementary food ranged from 7.13 ± 0.23/100 g to 13.31 ± 0.01/100 g, showing statistically significant results with *p* < 0.05. These results are aligned with the moisture content of Infant complementary food produced from malt rice, soybean, and pumpkin pulp flour, with the results reported as 8.0% and 10.9% (Anthony et al. 2020). These differences might be due to the quality of land cultivation, climatic conditions, processing methods, etc. The moisture content in all the formulated foods was lower than that of the carrot powder, confirming the influence of different raw materials blending. Among the formulations, OS_7_ had significantly lower moisture content (7.13 ± 0.03/100 g); this may be due to the inclusion of a moderate amount of Oat‐Teff mixture and Carrot powder (31.2 g), which shared a significant moisture contribution shown in (Table 2). According to WHO/FAO (2016), the recommended appropriate moisture for Infant complementary foods is less than 5%. However, in the present study, complementary foods were found to be above. This is because combining different foodstuffs used in the Infant food formulations can absorb more moisture.

##### 3.2.1.2 Ash

The ash content of the complementary food ranged from 2.76 ± 0.03/100 g to 3.40 ± 0.02/100 g, and it showed a closer agreement with the recommended levels of WHO (< 3%) and also agreed with earlier report (Roshana and Mahendran 2019). Points to the recommended levels of WHO, all the formulated products have a better quantity of minerals and follow the recommended level of WHO and CAC guidelines.

##### 3.2.1.3 Fiber

The prepared complementary foods showed the fiber content ranged from 2.67 ± 0.02/100 g to 12.11 ± 0.02/100 g. This is evidenced by increasing the amount of Carrot powder, which enhances the fiber content on OS_12_ and, followed by OS_11_, influences the fiber content positively. According to the WHO/FAO codex standard, the recommended value of fiber in complementary food should be less than 5%. Thus, many of the studied formulated composite products meet WHO/FAO recommended levels. In addition, products with lower fiber in complementary food lower the bulkiness of the food and boost protein digestibility. It ensures better nutrient absorption and minerals.

##### 3.2.1.4 Fat

The fat content of the formulations differs significantly (*p* < 0.05) and is reported in the range between 2.68 ± 0.02/100 g (OS_1_) and 4.27 ± 0.02/100 g (OS_4_). The highest fat (4.27 ± 0.02/100 g) was noted in composites of 62.2 g (Teff and Oat), 27.8 g Carrot powder, and 10 g Jaggery (OS_4_) which is comparable with the fat content of Infant complementary food produced from malted pregelatinized maize, soybean, and Carrot flowers, with the result of 4.93 ± 0.19% (Obinna‐Echem et al. 2018). The lower fat content was found in OS_1_ (formulated with 65.6 g of the Teff‐Oat mixture, 24.2 g Carrot powder, and 10.2 g Jaggery). It shows increased fat content upon the increment of Oat flour proportions. Currently, studied results were slightly similar or lesser to some extent to the complementary food prepared from red Teff and QPM (quality protein maize), which reported fat from 4.68% to 5.74% (Beruk et al. 2015). Furthermore, many currently studied formulated composite products meet WHO/FAO recommended levels.

According to FAO/WHO Codex CAC/GL 08 (2018), the crude fat content of complementary food should be between 10% and 25%. However, currently studied formulated composite foods are lower; this might be due to the absence of fat‐rich materials in the studied formulations.

##### 3.2.1.5 Protein Content

The presently studied complementary foods have protein content in the range of 9.89 ± 0.02/100 g to 15.97 ± 0.03/100 g, which corresponds to the formulations OS_1_ and OS_7_, which contain 27.9% Teff, 27.9% Oat, 31.2% Carrot powder, and 13% Jaggery (OS_1_) in range with the result of protein contents ranged from 0.16% to 16.87% (Onwuchekwa et al. 2025). In this case, the amount of Oat is high (i.e., 29.35%), which helps enhance the amount of protein. The amount of protein in many of the prepared composite complementary foods is the standard WHO recommends for complementary infant foods. According to the Codex Alimentarius Commission guideline, the food samples must have greater than 15 g/100 g of protein. Thus, the germinated cereals of Teff and Oats show a vital role in enhancing the protein in the presently studied formulated complementary food, which has a positive relation with the recommended level (shown in Table 2).

##### 3.2.1.6 Available Carbohydrates

The formulated food samples have reported significant differences (*p* < 0.05) with carbohydrates ranging from 54.87 ± 0.02 g/100 g in OS_12_ to 69.34 ± 0.03 g/100 g in OS_1_ (shown in Table 2). This is due to the more significant contribution of Oat (32.8 g/100 g from the Oat‐Teff mixture) in OS_1_ than in other ingredients of composite flour. Studied complementary food samples were more significant than those reported earlier (Ademulegun and Koleosho 2012), from 53.95 ± 0.02 to 61.73 ± 0.09. In comparison, lower carbohydrates (54.87 ± 0.02/100 g) were reported in the prepared complementary food: OS_12_. However, according to the UICEC/WHO (2005), the presently studied food samples contained higher amounts of carbohydrates than the requirement (37%) to fulfill daily consumption (Scherz and Bonn 2008; Lagnika et al. 2019). The recommended carbohydrate level as per CODEX CAC/GL was 60%–75% for complementary foods, which aligns with the currently studied complementary foodstuff. Hence, the amount of carbohydrates in all the prepared composite food flours is at the recommended level.

##### 3.2.1.7 Gross Energy

As seen results in Table 2, energy content (in kcal/100 g) was calculated from their fat, proteins, and carbohydrates, which were noticed as significantly different (*p* < 0.05). The formulated complementary food samples provide energy ranging from 301.62 ± 0.02 kcal/100 g to 360.47 ± 0.03 kcal/100 g. The result shows that combining Oat‐Teff, Carrot powder, and Jaggery influenced the energy value compared with all prepared foods (Table 2). Accordingly, the results obtained from the food mixture OS_3_ showed a higher energy value. It is supported by the higher amount of Oat‐Teff mixture, which contributes to more fat and protein and influences the energy increment of formulated complementary food, OS_3_. Also, the present results reveal that all the studied formulated food samples agree with the Codex standard recommendation (320–375 kcal/100 g) for complementary Infant foods.

#### 3.2.2 Functional Properties of Complementary Foods for Optimization

The functional properties such as swelling index, protein solubility index, bulk density, water absorption capacity, and oil absorption capacity of the formulated foods were analyzed, and the obtained results were presented in Table 3. The prepared complementary foods' swelling index differed significantly (*p* < 0.05) in the range between 2.64 ± 0.01 mL/g and 3.77 ± 0.02 mL/g (shown in Table 3). The swelling index of the product OS_7_ is reported as higher (3.77 ± 0.02 mL/g) with the composite flour containing a higher amount of Oat‐Teff. Compared to the earlier report, the swelling index of the presently studied formulated foodstuffs was lower in the range of 6.39–6.84 mL/g. Swelling capacity may be lower, which is required in complementary feeding as it increases the nutrient density of the food. It seems the child can consume more to meet the nutrient requirement (Olaitan et al. 2014). Accordingly, the composite flour OS_9_ was reported as a better nutrient with a lower swelling capacity (2.64 ± 0.01 mL/g).

**TABLE 3:** Functional properties of prepared composite complementary foods.

Samples blending ratio, g				Functional properties of formulated composite foods				
Code	A	B	C	SI (mL/g)	PSI (mL/g)	BD (g/mL)	WAC (g/mL)	OAC (g/g)
OS_1_	65.6	24.2	10.2	3.36 ± 0.02^e^	0.52 ± 0.03^a^	0.69 ± 0.02^gh^	2.23 ± 0.02^i^	3.52 ± 0.02^cde^
OS_2_	63.0	26.3	10.7	2.88 ± 0.02^h^	0.52 ± 0.02^a^	0.73 ± 0.02^fg^	2.52 ± 0.03^fg^	3.22 ± 0.02^fg^
OS_3_	63.7	25.0	11.3	3.66 ± 0.02^b^	0.42 ± 0.02^bc^	0.77 ± 0.02^def^	3.23 ± 0.03^d^	2.71 ± 0.02^h^
OS_4_	62.2	27.8	10.0	3.10 ± 0.05^h^	0.34 ± 0.02^d^	0.74 ± 0.01^efg^	3.42 ± 0.02^c^	4.12 ± 0.02^b^
OS_5_	58.1	30.0	11.9	3.23 ± 0.02^f^	0.23 ± 0.03^e^	0.78 ± 0.01^cde^	1.91 ± 0.02^j^	3.63 ± 0.02^c^
OS_6_	61.9	25.7	12.4	2.75 ± 0.03^i^	0.37 ± 0.03c^d^	0.71 ± 0.01^g^	2.33 ± 0.02 h^i^	3.33 ± 0.02^efg^
OS_7_	55.8	31.2	13.0	3.77 ± 0.02^a^	0.54 ± 0.02^a^	0.83 ± 0.02^ab^	1.92 ± 0.02^j^	2.12 ± 0.02^j^
OS_8_	54.1	32.5	13.4	3.40 ± 0.01^de^	0.43 ± 0.03^bc^	0.80 ± 0.02^abcd^	3.12 ± 0.03^d^	4.51 ± 0.04^a^
OS_9_	51.2	35.0	13.8	2.64 ± 0.01^j^	0.57 ± 0.02^a^	0.82 ± 0.0^abc^	3.82 ± 0.02^b^	1.80 ± 0.02^k^
OS_10_	52.4	33.1	14.5	3.67 ± 0.02^b^	0.26 ± 0.02^e^	0.85 ± 0.02^ab^	2.63 ± 0.02^f^	1.97 ± 0.02^jk^
OS_11_	50.6	34.2	15.2	3.45 ± 0.02^d^	0.35 ± 0.02^d^	0.85 ± 0.03^a^	1.75 ± 0.02^k^	2.33 ± 0.02^i^
OS_12_	57.4	26.6	16.0	3.57 ± 0.02^c^	0.55 ± 0.02^a^	0.77 ± 0.01^def^	2.78 ± 0.03^e^	3.42 ± 0.02d^ef^
OS_13_	58.7	28.5	12.8	3.25 ± 0.03^f^	0.53 ± 0.02^a^	0.74 ± 0.01^efg^	2.42 ± 0.02^gh^	3.57 ± 0.15^cd^

*Note:* Expressed the results as in mean ± SD; Means not shared by the same superscript letter are signigicantly different (*p* < 0.05); A: 1:1 oat/teff mixture (g); B: amount of carrot powder (g); C: amount of jaggery (g).

Abbreviations: BD, Bulk density; OAC, oil absorption capacity; PSI, protein solubility index; SI, swelling index; WAC, water absorption capacity.

The protein solubility index (PSI) is a protein in water used widely in the food industry. The PSI correlates directly with digestibility and protein availability. PSI also depends on the relative amount of polar and nonpolar amino acids, their three‐dimensional protein arrangement, and their interaction with water (Capriţa et al. 2010). Comparatively lower and higher values of the protein solubility index were found in composites of OS5 (0.23 ± 0.03 mL/g) and OS9 (0.57 ± 0.02 mL/g). It indicates that composite flour OS9 has a higher Carrot powder (35%), which supplies more soluble proteins. It proposes an enhancement in the nutritional quality of composite food through blending with other flours supported by earlier literature (Nawaz et al. 2015).

Bulk density (BD) is essential for material handling processing (during packaging and transportation) and application in food industries. Rashida et al. (2014) stated that lower bulk density could be advantageous in formulating complementary foods for Infants. The studied formulations' bulk density was in the range of 0.69 ± 0.02–0.85 ± 0.03 g/mL. It shows a good agreement with the result obtained from the formulation of complementary foods, which was developed by blending cereal, oilseed, and animal polypeptide, which showed that the results are in the range between 0.61 ± 0.01 and 0.69 ± 0.01 g/mL. According to Rashida et al. (2014), low BD of flour for Infant food preparation but higher amounts of flour particles lead to which can stay together, thus increasing such diets' energy. Due to this, more samples might be prepared using a lower amount of water with the desired nutrient energy density and semisolid consistency, which both Infants and adults can easily consume. Hence, the study results reported that formulated sample OS_1_ (containing 32.8 g, 32.8 g, 24.2 g, and 10.2 g of Teff, Oat, Carrot powder, and Jaggery, respectively) was considered a better Infant formulation with low BD (0.69 ± 0.02 g/mL). The low BD value may contribute to the higher Oat and Teff (32.8 g/100 g of each) in the OS_1_ formulation.

The water absorption capacity (WAC) is directly associated with the food products' microbial and shelf‐life (Ademulegun and Koleosho 2012). The WAC of the prepared food samples was found to differ significantly (*p* < 0.05), which ranged between 1.75 ± 0.02 (OS_11_) and 3.82 ± 0.02 g/mL (OS_9_). Generally, Porridges were used as complementary foods to be neither thick nor thin, which provide less energy and nutrient density to the Infants. So, lower WAC is appropriate in complementary food for making thinner gruels of porridges with high caloric density per unit volume, which is a better complementary food with lower WAC for infant formulation. Hence, all the studied food samples were appropriate complementary food to supply high calories to the Infants and any age holders.

The oil absorption capacity (OAC) of composite flours is an essential functional quality for assessing the sensory properties of food products, and it is noticeable that high OAC is necessary for improving the energy of balancing foods. As seen in the results in Table 3, the OAC of the formulated foods resulted (with *p* < 0.05) in the range from 11.80 ± 0.02 to 4.51 ± 0.04 g/g. This may be due to the different composition and processing conditions of complementary food currently being studied. Food products with higher protein content show high oil absorption. It may be due to proteins containing different nonpolar side chains that bind hydrocarbon chains, thereby increasing oil absorption. In this context, the formulation OS_8_ reported a better protein (14.56 ± 0.12 g/100 g) with a maximum OAC of 4.51 ± 0.04 g/100 g (Table 3).

#### 3.2.3 Optimization of Complementary Food Formulations Through Minerals

Studied the common metals (Na, K, Ca, Fe, Zn) of foodstuff (raw materials), formulations, and obtained results given in Table 4. As seen, the results of all minerals are statistically significant with *p* < 0.05. It discloses that, similar to the ash content, the corresponding minerals varied in the foodstuffs studied. Commonly, the mineral composition of any foodstuff may influence food composition and the processing method (Robin 2017).

**TABLE 4:** Mineral content of oat/teff‐carrot powder‐jaggery composite complementary food samples.

Samples blending ratio, g				Mineral contents of formulated composite foods (mg/100 g)				
Code	A	B	C	Potassium	Sodium	Calcium	Iron	Zinc
OS_1_	65.6	24.2	10.2	112 ± 0.02^g^	11.3 ± 0.02^g^	20 ± 0.02^c^	10.5 ± 0.02^bc^	2.8 ± 0.00^b^
OS_2_	63.0	26.3	10.7	154 ± 0.03^b^	15.3 ± 0.02^b^	27 ± 0.03^b^	13.2 ± 0.02^a^	0.9 ± 0.0^fg^
OS_3_	63.7	25.0	11.3	135 ± 0.02^e^	13.0 ± 0.02^ef^	19 ± 0.01^c^	10.9 ± 0.01^bc^	3.4 ± 0.00^a^
OS_4_	62.2	27.8	10.0	145 ± 0.02^c^	13.1 ± 0.0^de^	38 ± 0.00^a^	9.6 ± 0.02^cd^	1.0 ± 0.01^def^
OS_5_	58.1	30.0	11.9	146 ± 0.01^c^	13.2 ± 0.01^de^	25 ± 0.01^b^	10.4 ± 0.03^bc^	3.0 ± 0.01^b^
OS_6_	61.9	25.7	12.4	143 ± 0.02^cd^	11.3 ± 0.02^g^	20 ± 0.02^c^	8.8 ± 0.03^f^	2.9 ± 0.02^b^
OS_7_	55.8	31.2	13.0	139 ± 0.02^de^	14.4 ± 0.02^c^	8.0 ± 0.01^f^	4.2 ± 0.02^g^	0.8 ± 0.00^fg^
OS_8_	54.1	32.5	13.4	138 ± 0.03^de^	13.5 ± 0.01^d^	18 ± 0.01^c^	6.2 ± 0.01^de^	1.4 ± 0.01^c^
OS_9_	51.2	35.0	13.8	157 ± 0.02^ab^	12.6 ± 0.01^f^	18 ± 0.02^c^	7.2 ± 0.18^de^	0.9 ± 0.0^fg^
OS_10_	52.4	33.1	14.5	127 ± 0.01^f^	11.2 ± 0.01^g^	10 ± 0.00^def^	9.8 ± 0.01^b^	1.0 ± 0.00^ef^
OS_11_	50.6	34.2	15.2	142 ± 0.01^cd^	13.5 ± 0.01^d^	9.0 ± 0.00^ef^	5.1 ± 0.00^h^	1.3 ± 0.02^cd^
OS_12_	57.4	26.6	16.0	129 ± 0.01^f^	13.4 ± 0.02^de^	13 ± 0.03^de^	5.4 ± 0.01^h^	0.9 ± 0.00^fg^
OS_13_	58.7	28.5	12.8	142 ± 0.01^cd^	13.3 ± 0.02^de^	13 ± 0.01^d^	6.9 ± 0.01^e^	0.7 ± 0.0^g^

*Note:* Expressed the results as in mean ± SD; Means not shared by the same superscript letter are significantly different (*p* < 0.05); A: 1:1 oat/teff mixture; B: the amount of carrot powder; C: the amount of jaggery.

##### 3.2.3.1 Sodium

The exchange of sodium with potassium across the cell membrane generates an energy gradient essential for nutrient absorption via membrane transport mechanisms. It is crucial in transmitting action potentials in nerve and muscle cells (Wack et al. 1997). The Infant Formulae Directive Association (IFDA) sets for Infant formulae have less sodium of 20 mg/100 g and more of 60 mg/100 g (Wack et al. 1997). Comparatively, formulation OS_2_ has 15.3 ± 0.02 mg/100 g (Table 4), higher in sodium among the formulations, and could reach the minimum sodium requirement to supply the Infants' needs.

##### 3.2.3.2 Potassium

The IFDA sets a minimum of 60 mg/100 kcal and a maximum of 145 mg/100 kcal of potassium (Wack et al. 1997). In the current study, prepared foods with potassium ranged between 112 ± 0.02 mg/100 g (in OS_1_) and 157 ± 0.02 mg/100 g (in OS_9_). Sample OS_9_ shows the highest potassium (157 ± 0.02 mg/100 g), followed by OS_2_ (154 ± 0.03 mg/100 g). This may be due to the inclusion of (35%) Carrot powder in the formulation (OS_9_). At the same time, the lower amount of potassium resulted in the formulation OS_1_ (with 32.8% of Teff), which might be due to the blending of lower potassium‐containing cereal (Teff) used in the OS_1_ formulation. Meanwhile, as currently studied, all food formulations fulfill the recommended potassium levels for Infant food formula as per the recommendation of IFDA.

##### 3.2.3.3 Calcium

The amount of calcium reported was 8.0 ± 0.01 mg/100 g to 38 ± 0.00 mg/100 g in the formulations, showing statistical significance (*p* < 0.05) upon the formulations. The formulation OS_2_ (which has a more significant amount of Teff and Oat: 31.5 g each) showed a higher amount of Ca (Table 4), though the lowest Ca in OS_7_ (which has 27.9 g of Teff and 27.9 g of Oat). It indicates that the amount of calcium directly correlates with Teff, especially, that is, the incrementing of Teff‐influenced and enhanced calcium in formulated foodstuffs (Chukwu et al. 2014). Teff contains better calcium (40 ± 0.01 mg/100 g) than other raw materials. CAC/GL 08 (2016) report stated that complementary foods should contain at least 250 mg/100 g of calcium. However, the results of the formulated products have lower Ca than the recommended level.

##### 3.2.3.4 Iron

Iron content in the presently studied composite foods ranged from 4.2 ± 0.02 mg/100 g to 13.2 ± 0.02 mg/100 g. The maximum iron content (13.2 ± 0.02 mg/100 g) in sample OS_2_ was found in formulation mixed (in g) with 31.5:31.5:26.3:10.2 portions of Teff, Oat, Carrot powder, and Jaggery, respectively. In contrast, sample OS_7_ has the lowest iron (4.2 ± 0.02 mg/100 g), which contains (in g) 27.9:27.9:31.2:13 of Teff, Oat, Carrot powder, and Jaggery. The experimental results were significantly different (*p* < 0.05) by varying the proportions of the blending mixture of food components. Results in the present study were higher than the previous report (Omolara and Victor 2017), which said the Fe content was between 3.96 ± 0.01 mg/100 g and 4.58 ± 0.01 mg/100 g. Furthermore, the results of most of the formulated composite samples agreed with the recommended iron level, ranging from 7 to 30 mg/100 g (CAC/GL).

##### 3.2.3.5 Zinc

Zinc content presently studied in prepared complementary foods was found to be from 0.7 ± 0.00 mg/100 g to 3.4 ± 0.01 mg/100 g, as shown in Table 4, which shows the significant differences among the formulations. The value of the products (OS_1_, OS_3_, OS_5_ & OS_6_) was greater than the result (2.6 mg/100 g) reported by Zhang et al. (2015). Also, FAO/WHO (2018) CODEX CAC/GL recommended that complementary foods contain 3.2 mg of Zn per 100 g of food samples. Accordingly, the formulation OS_3_ of this study achieves the FAO/WHO standards requirement by reporting the Zn content at 3.4 ± 0.01 mg/100 g.

#### 3.2.4 Optimization of Complementary Foods by β‐Carotene, Phytate, and Oxalates

The current study presented the β‐carotene, phytate, and oxalate contents of prepared composite food shown in (Table 5).

**TABLE 5:** β‐carotene, phytates, oxalates (per 100 g) of complementary foods, and bioavailability of minerals to phytate content.

Food mixture ratio (g)				β‐carotene (mg)	Phytate (mg)	Oxalate (mg)	Bioavailability of minerals (phytate: minerals)			
Code	A	B	C				Phytate: Fe	Phytate: Ca	Phytate: Zn	Phytate: Na
OS_1_	65.6	24.2	10.2	4.6 ± 0.01^k^	16.33 ± 1.53^l^	42.47 ± 0.25^i^	0.0547^b^	0.0744^d^	0.0170^b^	0.0242^g^
OS_2_	63.0	26.3	10.7	5.2 ± 0.00^h^	17.40 ± 0.66^k^	45.58 ± 0.02^e^	0.0642^a^	0.0942^b^	0.0051^d^	0.0306^a^
OS_3_	63.7	25.0	11.3	6 ± 0.03^j^	17.52 ± 0.10^j^	38.63 ± 0.15^l^	0.0526^c^	0.0658^f^	0.0192^a^	0.0258^e^
OS_4_	62.2	27.8	10.0	6.2 ± 12^g^	18.59 ± 0.08^e^	46.33 ± 0.15^d^	0.0437^f^	0.1241^a^	0.0053^d^	0.0245^g^
OS_5_	58.1	30.0	11.9	9.2 ± 0.01^f^	17.53 ± 0.03^j^	48.73 ± 0.15^c^	0.0502^d^	0.0866^c^	0.0169^b^	0.0262^d^
OS_6_	61.9	25.7	12.4	6 ± 0.05^i^	17.43 ± 0.25^gh^	49.55 ± 0.18^b^	0.0427^g^	0.0697^e^	0.0165^b^	0.0226^i^
OS_7_	55.8	31.2	13.0	7.3 ± 0.23^e^	18.63 ± 0.15^d^	44.77 ± 0.16^f^	0.0191^l^	0.0261^m^	0.0043^f^	0.0269^b^
OS_8_	54.1	32.5	13.4	7.1 ± 0.05^d^	18.45 ± 0.23^f^	40.32 ± 0.02^k^	0.0284^j^	0.0592^g^	0.0075^c^	0.0255^e^
OS_9_	51.2	35.0	13.8	8.3 ± 0.26^b^	19.32 ± 0.02^a^	33.53 ± 0.02^o^	0.0315^i^	0.0566^h^	0.0046^e^	0.0227^i^
OS_10_	52.4	33.1	14.5	7.1 ± 0.60^d^	18.20 ± 0.03^cd^	43.25 ± 0.02^h^	0.0456^e^	0.0334^k^	0.0054^d^	0.0214^j^
OS_11_	50.6	34.2	15.2	7.3 ± 0.05^c^	17.67 ± 0.15^hi^	50.04 ± 0.02^a^	0.0244^k^	0.0309^l^	0.0073^c^	0.0266^c^
OS_12_	57.4	26.6	16.0	6.5 ± 0.17^h^	18.83 ± 0.03^c^	35.82 ± 0.03^m^	0.0243^k^	0.0419^j^	0.0047^e^	0.0248^f^
OS_13_	58.7	28.5	12.8	5.7 ± 0.02^g^	17.50 ± 0.03^fg^	41.60 ± 0.02^j^	0.0334^h^	0.0455^i^	0.0039^g^	0.0265^c^

*Note:* Means not shared by the same superscript letters in the column is significantly different (*p* < 0.05); A: 1:1 oat/teff mixture; B: the amount of carrot powder; C: the amount of jaggery.

##### 3.2.4.1 β‐Carotene

The amount of β‐carotene in currently studied complementary food samples ranged between 4.6 ± 0.01 mg/100 g and 9.2 ± 0.01 (mg/100 g). Among all formulations, the results noticed a higher amount of β‐carotene in formulation OS_9_ by mixing 51.2 g (Oat‐Teff mixture), 35 g Carrot powder, and 13.8 g Jaggery. It is influenced mainly by the higher quantity of Carrot powder in the OS_9_ formula than in other formulations. These findings agree with the earlier study and WHO recommended level (Beruk et al. 2015; WHO/FAO 2016). Thus, the present study results confirm that the prepared composite formulations can efficiently supply the β‐carotene to the Infants.

##### 3.2.4.2 Phytates

Anti‐nutrients are natural or synthetic compounds that interfere with the absorption of nutrients. The amount of phytate in the formulated foods was analyzed and presented in Table 5. Phytate or 24 phytic acids are naturally occurring compounds in plants, and they were present in 25 different foodstuffs at 0.1 to 6.0% (Gupta et al. 2006). Mrinal et al. (2020) stated that anti‐nutrients are negatively charged constituents; they could drag the positively charged metal ions like iron, calcium, zinc, and sodium, reducing the bioavailability of those minerals.

Table 5 shows the higher phytate (19.32 ± 0.02 mg/100 g) in formulation OS_9_ and the lower phytate (16.33 ± 1.53 mg/100 g) in a composite blend of OS_1_. Tesfaye et al. (2020) reported the phytate content as between 64.64 mg and 102.57 mg. This might be due to the use of many kinds of cereals and legumes that contain different anti‐nutrients and follow other processing methods like soaking, fermentation, and germination that help reduce the anti‐nutrient content of foods. Comparatively, the composite food samples have reported lower phytate; this may be the effect of germinated Oat and Teff in the present study prepared composites. In the current study, composite flours were fortified with the lower phytate‐containing raw materials (Carrot powder and Jaggery).

Generally, for Infants and all customers, foods containing lower phytate are recommended. But then, still, the phytate content of the currently formulated composites was showing more inferior to the recommended level compared to the daily intake of phytate from complementary foods (300–500 mg/day), which is recommended as per the WHO guideline (2016). The reported results in the current study suggest that all the studied composites of formulated complementary foods were well‐suited to Infants and middle‐aged (12–24 month) holders.

The bioavailability of minerals concerning phytate was analyzed, and the results are presented in Table 5. The results show that the higher phytate: Ca and phytate: Fe ratios were reported than phytate: Na and phytate: Zn in all formulations, indicating lower Ca and Fe bioavailability, respectively. Because of its maximum concentration and robust affinity for phytate, the divalent Ca ion may utilize a sparing action for Fe and Zn by forming phytate–Ca complexes (Dahiya et al. 2014). However, in all cases, the phytate: minerals ratio was reported lower than one. Hence, in all the studied formulations, in vitro Ca, Na, Fe, and Zn accessibility was unaffected by phytate (Table 5). So, all formulated complementary Infant foods have enough minerals required for the body's metabolism.

##### 3.2.4.3 Oxalates

Oxalates were found primarily in different plant products. Like phytate, other anti‐nutrients of oxalates can also form an insoluble salt with metal ions. It may be dangerous because reacting with calcium is not dissolved in an acidic environment in the human body, leading to kidney stone problems. The oxalate content of currently studied prepared food samples ranged from 33.53 ± 0.02 mg to 50.04 ± 0.02 mg, and the results showed significantly different with *p* < 0.05. Currently formulated products found lower oxalate content than the result reported by Akshay et al. (2015), which showed 180–225 mg/100 g. Foods that contain more oxalate, which causes lower calcium, are considered toxic for humans. According to the American Dietetic Association (Maillet et al. 2005), the oxalate intake recommendation is 40–50 mg/day. Thus, oxalate contents in all of the formulated food samples and raw materials are at a safer level for consumption by Infants and all age‐holders.

#### 3.2.5 Optimization of Complementary Food Formulations Using an Overlaid Contour Plot

According to Manary et al. (2004), the white ‘sweet spot’ appears in contour plots that optimize the responses by revealing the level of food components in the prepared composite foods. It could be found to be fixed by keeping the lower and upper constraints of responses obtained by the researchers. Commonly, for optimization, responses that show significant differences were considered. So, this current study used the statistically significant (*p* < 0.05) food constituents of nutrients (proximate components and minerals), anti‐nutrients, and physicochemical properties to optimize the region through contour plot design.

**FIGURE 1:**
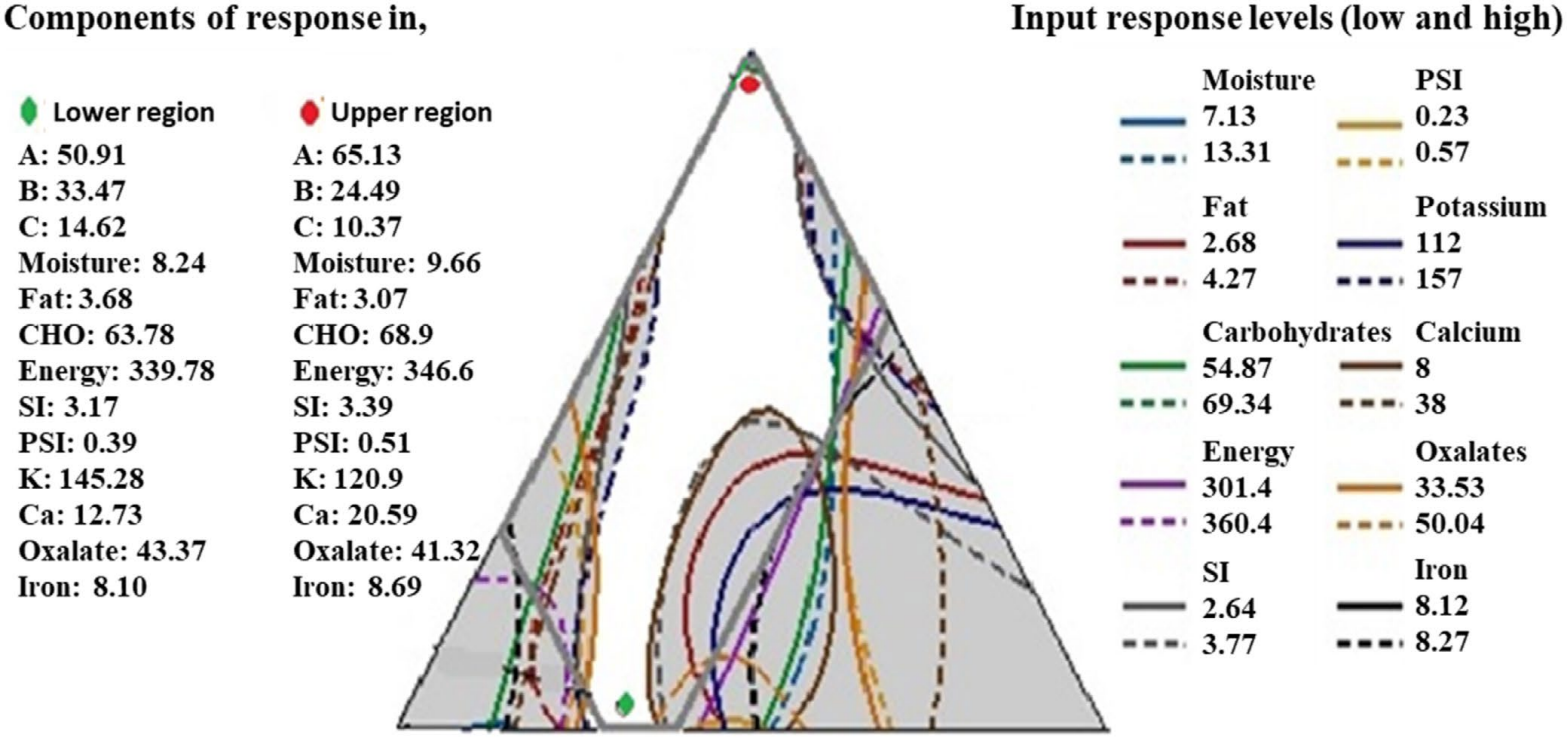
Overlaid contour plots for Carrot and Jaggery blended with Teff‐Oat mixture, which contains moisture, fat, carbohydrates, energy, swelling index, protein solubility index, K, Ca, oxalate, and pH; the white area shows the ‘sweet spot’ that optimizes the response variables listed in the respective legends.

Optimization does follow regarding the significant values of the analyzed food characteristics. Figure 1 established the overlaid contour plots through different combinations of significant responses of food attributes by using the lower and upper bounds obtained in the study. The obtained overlaid contour plot (Figure 1) has a white sweet spot, which shows all the combinations of significant parameters contribute an influential role in selecting the optimum composition (Aynalem and Ramesh 2022) of the composite (with the corresponding input of responses) to make a better complementary composite food. Furthermore, the studied contour plot shows better blending ratios of the food components between the Teff, Oat, Carrot powder, and Jaggery composite.

The overlaid contour plot design was made in Figure 1 by employing the significant value of *p* < 0.05 responses: proximate compositions, functional properties, minerals, and oxalate to find the optimization region. Accordingly, the optimum region (lower and higher level) of the prepared composite food mixture was found as component A: 50.91 g–65.31 g (an equal mixture of Teff and Oat), B: 24.49 g–34.47 g (Carrot powder), and C: 9.66 g–14.62 g (Jaggery) with the corresponding optimum level of responses (moisture: 8.24 g/100 g–9.62 g/100 g; fat: 3.01 g/100 g–3.71 g/100 g; CHO: 63.71 g/100 g–68.96 g/100 g; energy: 340.05 kcal/100 g–346.18 kcal/100 g; swelling index: 3.15 mL/g–3.44 mL/g; protein solubility index: 0.39 mL/g–0.51 mL/g; K: 119.29 mg/100 g–146.02 mg/100 g; Ca: 13.02 mg/100 g–21.01 mg/100 g; pH: 6.38–6.54; oxalate: 41.39 mg/100 g–43.24 mg/100 g); overall acceptability: 5.87–6.51 (under 7‐point hedonic scale) was found from the overlaid contour plot (shown in Figure 1).

#### 3.2.6 Optimization of Variables Using Response D‐Optimizer

The present study used mixture components with special cubic model terms and employed the mixture regression approach. Based on the D‐optimizer composite desirability, four better predictions of optimum food blending proportions were provided (as shown in Table 6). It reveals the predicted mixer ratios ranged (Teff/Oat: 55.62 g/100 g–65.37 g/100 g, Carrot powder: 24.2 g/100 g–32.96 g/100 g, and Jaggery: 10 g/100 g–14.82 g/100 g) with suitable responses of each quality parameter. The ratio of this study has begun the range of the optimization at experimental design (i.e., A: 50 g–65 g; B: 24 g–35 g; C: 10 g–15 g).

**TABLE 6:** Optimized variables (composite proportions and their quality parameters) using response D‐optimizer.

Optimized composite food coding	Food composition (g/100 g)			Predicted response values	The desirability for each response	Composite desirability
	A	B	C			
S_1_	55.62	32.96	11.42	Moisture (g/100 g): 9.24	0.7004	0.9318
				Fat (g/100 g): 4.13	0.9536	
				Energy (kcal/100 g): 348.36	0.8007	
				Swelling index (g/mL): 0.55	1.0000	
				Protein solubility index (g/mL): 0.55	1.0000	
				Potassium (mg/100 g): 166.54	1.0000	
				Oxalate (mg/100 g): 31.3	1.0000	
				Ca (mg/100 g): 37.1	1.0000	
S_2_	65.37	24.20	10.43	Carbohydrates (g/100 g): 69.52	1.0000	0.9552
				Energy (kcal/100 g): 348.61	0.8069	
				Protein solubility index (g/mL): 0.53	0.9408	
				β‐carotene (mg/100 g): 4.85	0.9642	
				Swelling index (g/mL): 3.37	1.0000	
				Oxalate (mg/100 g):40.20	1.0000	
				Iron (mg/100 g): 10.15	0.9294	
				Overall acceptability: 5.93	1.0000	
S_3_	60.43	24.75	14.82	Fat (g/100 g): 4.51	1.0000	0.9635
				Protein (g/100 g): 14.51	0.7683	
				Protein solubility index (g/mL): 0.54	0.9768	
				Swelling index (g/mL): 2.35	1.0000	
				Potassium (mg/100 g): 162.96	1.0000	
				Phytate (mg/100 g): 17.83	0.9995	
				Calcium (mg/100 g): 8.35	1.0000	
S_4_	59.50	30.50	10.00	Fat (g/100 g): 4.66	1.0000	0.9736
				Protein (g/100 g): 18.49	1.0000	
				Protein solubility index (g/mL): 0.40	0.8219	
				Swelling index (g/mL): 2.38	1.0000	
				β‐carotene (mg/100 g): 5.04	1.0000	
				Calcium (mg/100 g): 50.69	1.0000	
				Iron (mg/100 g): 8.27	0.9318	
				Phytate (mg/100 g): 17.92	0.9825	
				Oxalate (mg/100 g): 41.10	1.0000	
				Overall acceptability: 6.63	1.0000	

*Note:* A: 1:1 oat/teff mixture (g); B: amount of carrot powder (g); C: amount of jaggery (g).

The optimized range of food components was almost similar to the assumption (10–15 g). Hence, it proved the assumption of this study through the overlaid contour plot “white sweet spot” region (Figure 1). It indicates that the studied components of the composite mixture are well‐fitted (interacted) to formulate the food formula. Furthermore, the blended flour component proportions with the predicted responses, as suggested by the D‐optimizer (shown in Table 6), agree with the levels predicted from the contour plot (Figure 1).

All four composite component mixtures were selected (for further optimization) to achieve higher composite desirability (> 0.90). Then, all those four optimized food component mixtures were formulated separately (coded: S_1_–S_4_), as shown in Table 6. Then, these optimized food samples were assessed through their sensory characteristics. The results were compared with those of the control sample (Mothers' choice, commercial Infant food).

### 3.3 Sensory Evaluation of Optimized Composite Foods and Control

**FIGURE 2:**
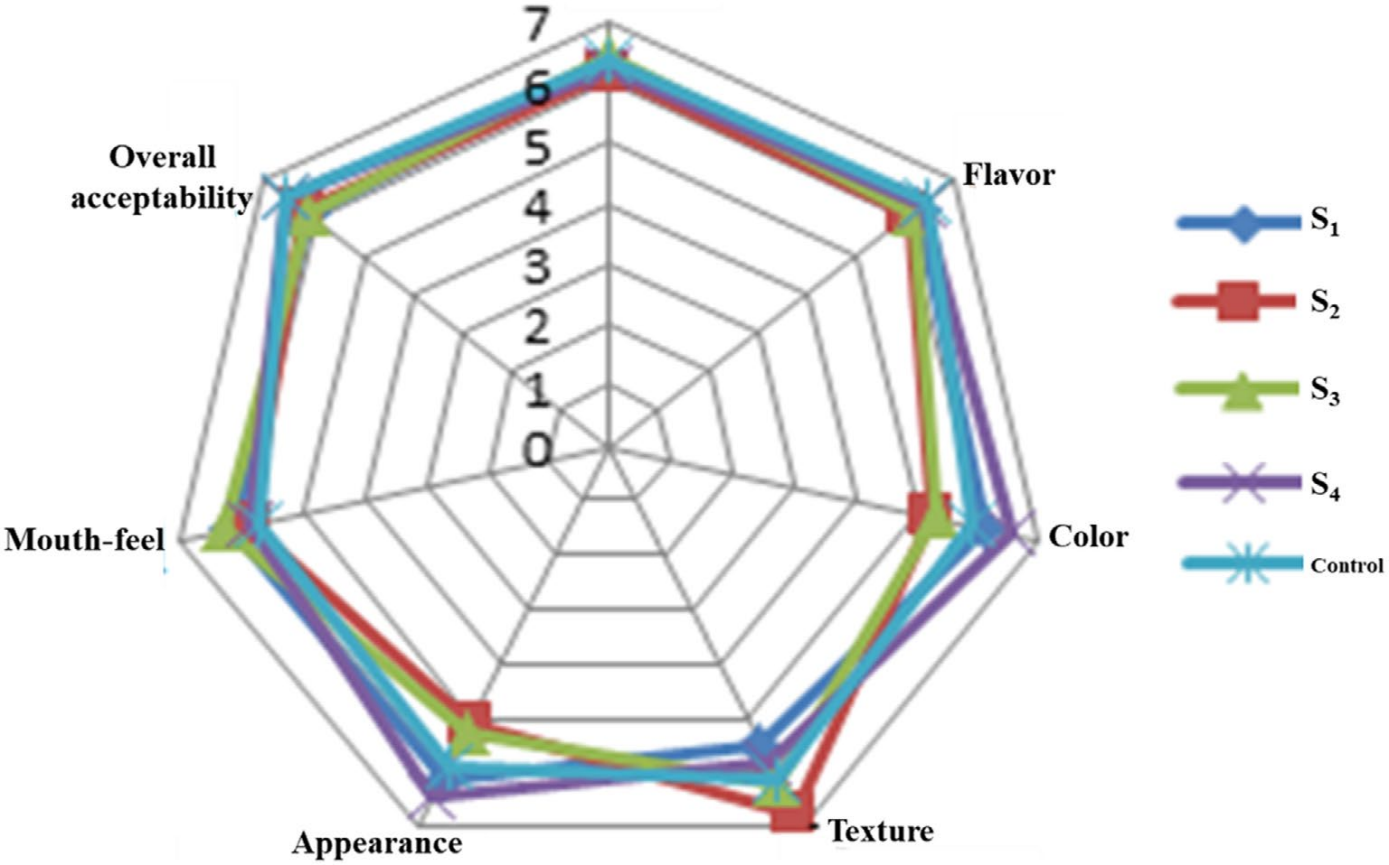
Web diagram for sensory analysis of composite foods and baby food (control) Infant food sample.

The scores of sensory attributes assessed (taste, flavor, color, texture, appearance, mouthfeel, and overall acceptability) were for the optimized, prepared food formulations of porridges using a seven‐point hedonic scale by semitrained panelists, which were described in a web diagram (Figure 2).

Taste is an essential sensory attribute for the recognition of food products. A higher web diagram point for taste was recorded in S_3_ (6.44 ± 0.13), containing more Jaggery, which may supply the sweetening. Also, the taste score of the prepared samples ranged from 6.13 ± 0.20 to 6.44 ± 0.13, with a significant difference (*p* < 0.05). These scores are almost closer to the control sample. This indicates that all the prepared complementary foods taste like those in the control sample. So, the prepared samples are alternatives to the control sample and may prefer to be consumed by Infants.

Generally, the flavor is the whole impression of foodstuffs with odor, taste, and mouth feel. So, flavor is an important attribute when assessing the suitability of articulated foods. In the current study, the flavor score of the prepared optimized food samples was found to be significantly different (except S_4_) compared to their control (Mother's choice) Infant food (shown in Figure 2). The flavor score of fortified food samples ranges from 6.86 to 7.14 (out of a 9‐point hedonic scale), as reported earlier (Shahid et al. 2008), to be closer to the studied samples flavor score from 6.10 ± 1.02 to 6.39 ± 0.13 (out of a 7‐point hedonic scale). It is supported by the idea that the currently prepared optimized complementary foods show better flavor.

The color's mean score was from 5.24 ± 0.80 to 6.51 ± 0.04 in prepared optimized complementary foods with different proportions of optimized blends. As seen in the web diagram (Figure 2), the color scores of S1 and S4 are 6.08 ± 0.32 and 6.51 ± 0.04, higher than the control sample (color value: 5.89 ± 0.72). It may be because more Carrot powder is blended on those optimized foodstuffs than in the control sample. Furthermore, adding Carrot powder did not affect all the textural and organoleptic attributes of the optimized complementary foods compared to the control (Mother's choice) sample. Also, the food products' color value depends on physicochemical characteristics such as water content, sugars, and protein content. The optimized food sample (S_4_) has more protein with lower pH values than predicted (Table 5).

The prepared complementary foods texture score ranged from 5.53 ± 0.82 to 6.73 ± 0.52. Here, study results showed a lower texture score in the control sample, 6.15 ± 0.26, than in the S_2_ formulation (which contains more Teff/Oat combination), and the control sample had a nonsignificant difference with S_3_, as shown in Figure 2. However, the other three optimized formulations differed significantly (*p* < 0.05) compared to the control sample. Thus, most of the studied complementary food samples showed better texture than the control sample.

The appearance of any food covers all the visible attributes, such as shape, color, transparency, and density (Bello et al. 2020). Prepared optimized foods reported the appearance score range of 5.13 ± 0.26–6.47 ± 0.24, which is statistically significant; it shows better agreement with the earlier report (Bello et al. 2020). At the same time, this study revealed a significantly lower appearance (5.91 ± 0.08) in the control sample than in the prepared samples (S_1_, 6.13 ± 0.07 and S4, 6.47 ± 1.24). Hence, optimized complementary foods S_1_ and S_4_ are acceptable to fulfill the need for Infant formulations regarding appearance.

As with other sensory attributes, the mouthfeel is essential to credit food products. The highest score was 6.30 ± 0.15 (in S_3_), followed by other prepared optimized complementary foods, whereas the recorded minimum score was in the control (5.74 ± 0.15) with statistical significance (shown in Figure 2). This may be due to the availability of different proportions of Jaggery in the prepared sample, and there might be a small amount of sweetener in the control sample. It reveals that all the prepared complementary foods achieve a better mouthfeel than the control sample. So, the prepared samples are a substitute for the control sample and may be preferred to be consumed by Infants as a complementary food.

The overall acceptability score of the studied optimized sample S_4_ achieved a maximum score of 6.55 ± 0.58 and a good agreement (statistically nonsignificant) with the control sample. Furthermore, the results obtained in the present study (ranged from 6.11 ± 1.09 to 6.55 ± 0.58) are closer in agreement with the earlier study Veronika et al. (2018), which resulted in similar results concerning the overall acceptability score of sensory evaluation in soy flour fortified flour. Also, the USDA (2009) report supported the studied results, which reported that the fat levels ranging from 2% to 5% might improve the product's storability due to the lower chance of rancid flavor development Veronika et al. (2018). Hence, all the optimized complementary foods are in the range of recommended fat levels, which confirms that the prepared optimized complementary foods might have a longer shelf‐life. According to WHO/FAO (2016), all formulations were acceptable as they received overall acceptability scores greater than 4 (under a 7‐point hedonic scale), ranging from 6.11 ± 1.09–6.55 ± 0.58.

Therefore, considering all the food quality parameters and most sensory attribute scores, the present study concludes that the S_4_ (59.5 g (1:1, Teff/Oat mixture), 30.5 g Carrot powder, and 10 g of Jaggery) complementary composite food sample showed better optimized blended food composition between the studied components with the composite response desirability of 0.9736.

### 3.4 Validation of Better Optimized Product

In the current study, S_4_ is the best‐optimized product among the optimized complementary foods based on the studied food nutrients (shown in Table 5) and sensory attributes (shown in Figure 2). A freshly prepared formulation of composite flour, S_4_, determined its characteristics responses to display the best optimized complementary food.

Then, compare the obtained actual experimental response values (shown in Table 7) of S_4_ and commercially available Infant foods, namely Mother's choice and Cerifam (as control), with the predicted results (supported by the response D‐optimizer software) and WHO standard values (shown in Table 7). It reveals that the obtained results of S_4_ were closer to the predicted values. At the same time, the results of fat (4.78 ± 0.05 g/100 g), protein (18.27 ± 1.06 g/100 g), PSI (0.42 ± 0.02 mL/g), β‐carotene (4.87 ± 0.04 mg/100 g) of S_4_ are higher than commercial Infant foods, which is familiar in the Ethiopian market. They are within the WHO‐recommended nutrient intake level (Table 7).

**TABLE 7:** Validation of experimental results of sample S_4_ with its predicted commercial infant foods and WHO recommended nutrient intake.

Predicted responses	Predicted response values	Experimental response values			RNI/day by WHO
		Current study (S_4_)	Commercial infant foods (control samples)		
			(Mothers' choice)	Cerifam	
Fat (g/100 g)	4.66	4.78 ± 0.05	3.64 ± 0.27	4.51 ± 0.06	5–25
Protein (g/100 g)	18.49	18.27 ± 1.06	15.38 ± 0.05	15.32 ± 0.35	9.1–71
Swelling index (mL/g)	2.38	2.40 ± 0.27	5.92 ± 0.03	5.23 ± 0.04	—
PSI (mL/g)	0.40	0.42 ± 0.02	0.39 ± 0.18	0.34 ± 0.01	0.35–0.60
β‐carotene (mg/100 g)	5.04	4.87 ± 0.04	1.73 ± 0.09	0.32 ± 0.02	3–6
Ca (mg/100 g)	50.69	50.57 ± 0.02	105.20 ± 1.05	98.25 ± 0.26	250–1200
Fe (mg/100 g)	8.27	8.12 ± 1.47	8.38 ± 0.51	6.05 ± 0.18	7–30
Phytate (mg/100 g)	17.92	17.16 ± 0.06	34.8 ± 0.24	2.8 ± 0.52	Low
Oxalate (mg/100 g)	41.01	40.86 ± 0.01	17.53 ± 0.40	1.14 ± 0.08	40–50
Overall acceptability	6.63	6.55 ± 0.58	6.59 ± 0.31	6.62 ± 0.51	> 4

Abbreviations: PSI, protein solubility index; RNI, recommended nutrient intake; S_4_, 59.5 g Teff/Oat mixture +30.5 g carrot powder +10 g jaggery containing currently studied formulated infant food.

Presently studied experimental results of S_4_ complementary food show the amount of Ca and Fe and overall acceptability are 50.57 ± 0.02 mg, 8.12 ± 1.47, and 6.55 ± 0.58, respectively, which approach the predicted value. The amount of Fe and overall acceptability agree with control samples and the allowable limit of WHO standards. However, Ca is lower than (two times the control and five times lower than the WHO standard recommendation). This is because of the different compositional nature of foodstuffs (raw materials) used in the formulation, such as Teff, which has a higher amount of iron that contributes to the Fe of the composite flour. However, the calcium needs additional blending (if required) to achieve the recommended levels.

Better optimized product (S_4_) anti‐nutrient component (phytate and oxalates) is closer to the predicted response values. Also, the phytate and oxalates of S_4_ were lower and greater than those of the studied control Infant foods. Still, these anti‐nutrient values (shown in Table 7) are agreeable according to WHO standards (i.e., within the recommended limit) for formulating complementary Infant food to achieve nutrient needs. Hence, this study is expected as this level of anti‐nutrients might not retard the bioavailability of minerals according to the earlier study Hasan et al. (2014). Thus, the current study concluded and proposed the formulated better‐optimized complementary composite food (S_4_) for Infants and middle‐holders.

## 4 Conclusions

Infant malnutrition is a significant health problem in many developing countries, including Ethiopia, so it needs critical attention. Thus, malnutrition is reduced by providing proper nutrition, which improves the average growth and development of the Infant. This study works on the formulation of complementary food from readily available sources (Oat, Teff, Carrot powder, and sugarcane Jaggery) by using a simple way of processing to achieve the nutritional requirements of Infants and Children through different mixing ratios and commercially existing Infant food chosen as control.

Based on the results of responses, the optimized composite complementary food S4 (which contains 59.5 g of 1:1 mixture of Oat/Teff, 30.5 g of Carrot powder, and 10 g of Jaggery) is a better composite with a composite desirability of 0.9736, which is also evidenced through sensory evaluations. The level of protein, iron, β‐carotene, phytates, and oxalates achieved the WHO recommended required daily intake. The study concludes that blending different studied food sources improves the necessary food nutrients. Therefore, the analysis results indicate that the selected foodstuffs (raw materials) were effectively processed and formulated into complementary food with enhanced nutritional quality, making it suitable for Infant feeding and anyone interested in consuming a nutritious product. The better‐optimized complementary food had a good nutritional profile and lower anti‐nutrients than the World Health Organization's standard recommended daily intake level. Thus, the studied results conclude that the presently formulated composite food is promising, unique, and value‐added to the coarse cereals (Oat and Teff), Carrot, and Jaggery under the complementary composite food category processing market.

## Author Contributions

**Ramesh Duraisamy:** conceptualization (equal), data curation (lead), formal analysis (equal), methodology (equal), project administration (lead), software (lead), supervision (lead), writing – original draft (equal), writing – review and editing (lead). **Bahiru Bekele:** conceptualization (equal), data curation (equal), investigation (lead), methodology (lead), visualization (lead), writing – original draft (equal). **Belay Haile:** formal analysis (equal), software (equal), validation (equal). **Eyob Mulugeta:** data curation (equal), resources (equal). **Tanje Mada:** resources (equal), software (equal), validation (equal).

## Ethics Statement

The authors have nothing to report.

## Conflicts of Interest

The authors declare no conflicts of interest.

## Data Availability

The data that support the findings of this study are available on request from the corresponding author.

## References

[fsn370264-bib-0001] Ademulegun, T. I. , and A. T. Koleosho . 2012. “Effects of Processing on the Nutrients' Composition of Maize/Soya Complementary Food.” IOSR Journal of Pharmacy and Biological Sciences (IOSR‐JPBS) 4: 39–43.

[fsn370264-bib-0002] Agrawal, D. , A. Upadhyay , P. S. Nayak , and V. K. D. Krishi . 2013. “Functional Characteristics of Malted Flour of Foxtail, Barnyard, Little Millets.” Annals. Food Science and Technology 14, no. 1: 44–49.

[fsn370264-bib-0003] Ahmed, B. M. , R. A. Hamid , M. E. Ali , A. B. Hassan , and E. E. Babikaer . 2006. “Proximate Composition, Antinutritional Factors and Protein Fraction of Guar Gum Seeds Are Influenced by Processing Treatment.” Pakistan Journal of Nutrition 5, no. 5: 481–484. 10.3923/pjn.2006.481.484.

[fsn370264-bib-0004] Akshay, K. , R. Akshay , and D. Rahul . 2015. “Developing Sugarcane Feeding System for Jaggery Making Plants for Rural India.” International Journal of Science Technology & Management 4: 225–228.

[fsn370264-bib-0005] Alexandratos, N. , and B. Jella . 2012. “World Agriculture Towards: The 2012 Revision.” In ESA Working Paper No. 12‐03. Agricultural Development Economics Division, Food and Agriculture Organization of the United Nations. http://www.fao.org/3/a‐ap106e.pdf.

[fsn370264-bib-0006] Anthony, N. M. , W. A. Afolabi , E. M. S. Williams , B. Mayiza‐Dixon , and O. E. Babatunde . 2020. “Appraisal and Composition of Some Traditional Complementary Foods for Infant Nutrition in Sierra Leone.” International Journal of the Science of Food and Agriculture 4, no. 1: 73–79. 10.26855/ijfsa.2020.03.011.

[fsn370264-bib-0007] AOAC , ed. 2023. Official Method of Analysis of the Association of Official Analytical Chemists. 22nd ed. AOAC International.

[fsn370264-bib-0008] Assuncao, M. C. F. , I. S. Santos , J. D. A. Barros , D. P. Gigante , and C. G. Victora . 2007. “Effect of Iron Fortification of Flour on Anemia in Preschool Children in Pelotas, Brazil.” Revista de Saúde Pública 41, no. 4: 539–548. 10.1590/s0034-89102006005000031. 17589751

[fsn370264-bib-0009] Aynalem, E. G. , and D. Ramesh . 2022. “Formulation and Optimization of Complementary Food Based on Its Nutritional and Antinutritional Analysis.” International Journal of Food Science 2022: 1126031. 10.1155/2022/1126031. 36299560 PMC9592211

[fsn370264-bib-0063] Bello, A. A. , D. I. Gernah , C. C. Ariahu , and J. K. Ikya . 2020. “Physico‐Chemical and Sensory Properties of Complementary Foods from Blends of Malted and Non‐Malted Sorghum, Soybean and *Moringa oleifera* Seed Flours.” American Journal of Food Science and Technology 8, no. 1: 1–13. 10.12691/ajfst-8-1-1.

[fsn370264-bib-0010] Beruk, B. , A. Kebede , and K. Esayas . 2015. “Assessment of Knowledge and Practices on Complementary Food Preparation and Child Feeding at Shebedino, Sidama Zone, Southern Ethiopia.” International Journal of Food Science and Nutrition Engineering 5: 82–87.

[fsn370264-bib-0011] Bouhouch, R. , S. El‐Fadeli , M. Andersson , A. Aboussad , L. Chabaa , and C. Z. Zeder . 2016. “Effects of Wheat‐Flour Biscuits Fortified With Iron and EDTA, Alone and in Combination, on Blood Lead Concentration, Iron Status, and Cognition in Children: A Double‐Blind Randomized Controlled Trial.” American Journal of Clinical Nutrition 104: 1318–1326. 10.3945/ajcn.115.129346. 27733396

[fsn370264-bib-0012] Capriţa, R. , A. Capriţa , and L. Creţescu . 2010. “Protein Solubility as Quality Index for Processed Soybean.” Animal Science and Biotechnologies 43, no. 1: 375–378.

[fsn370264-bib-0013] Catherine, T. N. , H. M. John , M. Reddy , and N. Dorothy . 2015. “Optimized Formulation and Processing Protocol for a Supplementary Bean‐Based Composite Flours.” Food Science & Nutrition 3, no. 6: 527–538. 10.1002/fsn3.244. 26788294 PMC4708651

[fsn370264-bib-0014] Chinma, C. E. , I. C. Alemede , and I. G. Emelife . 2008. “Physicochemical and Functional Properties of Some Nigerian Cowpea Varieties.” Pakistan Journal of Nutrition 7: 186–190. 10.1016/j.foodres.2009.04.024.

[fsn370264-bib-0060] Chukwu, U. , J. I. Afolami , and O. T. Adepoju . 2014. “Chemical Composition of Locally Made Complementary Food Standard Recipes in Nigeria.” WEST African Journal of Foods and Nutrition 12, no. 2: 1–81.

[fsn370264-bib-0015] Corbo, M. R. , A. Bevilacqua , L. Petruzzi , F. P. Casanova , and M. Sinigaglia . 2014. “Functional Beverages: The Emerging Side of Functional Foods: Commercial Trends, Research, and Health Implications.” Comprehensive Reviews in Food Science and Food Safety 13, no. 6: 1192–1206.

[fsn370264-bib-0016] Dahiya, P. K. , M. J. R. Nout , M. A. van Boekel , N. Khetarpaul , R. B. Grewal , and A. Linnemann . 2014. “Nutritional Characteristics of Mung Bean Foods.” British Food Journal 116, no. 6: 1031–1046. 10.1108/BFJ-11-2012-0280.

[fsn370264-bib-0017] Dewey, K. G. , and K. H. Brown . 2003. “Update on Technical Issues Concerning Complementary Feeding of Young Children in Developing Countries and Implications for Intervention Programs.” Food and Nutrition Bulletin 24, no. 1: 5–18. 10.1177/156482650302400102. 12664525

[fsn370264-bib-0018] Dilip, A. P. , M. S. Jadhav , and C. A. Nimbalkar . 2017. “Techniques and Advances in Jaggery Processing: A Review.” Research Journal of Chemical and Environmental Sciences 5: 14–20.

[fsn370264-bib-0019] Elena, P. , F. Juana , M. V. Cristina , M. Diana , A. Belen , and R. Daniel . 2017. Optimization of Germination Conditions by Response Surface Methodology to Obtain Oat Healthy Products. Submitted in Euro Food Chem XIX Conference, Spain. http://hdl.handle.net/10261/172371.

[fsn370264-bib-0020] Fardet, A. 2015. “Complex Foods Versus Functional Foods, Nutraceuticals and Dietary Supplements.” Agro Food Industry Hi‐Tech 26, no. 2: 20–24.

[fsn370264-bib-0021] Felistus, C. , C. Wisdom , A. Aggrey , S. John , M. Nzola , and M. Jonathan . 2017. “Analysis of Micronutrients Variations Among Sweet Potato (*Ipomoea Batatas Lam*) Genotypes in Malawi.” Journal of Agricultural Biotechnology and Sustainable Development 9, no. 4: 22–35.

[fsn370264-bib-0022] Gibson, G. R. , and C. M. Williams . 2000. Functional Foods: Concept to Product. 1st ed. Woodhead Publishing.

[fsn370264-bib-0023] Gibson, R. S. , K. B. Bailey , M. Gibbs , and E. L. Ferguson . 2010. “A Review of Phytate, Iron, Zinc, and Calcium Concentrations in Plant‐Based Complementary Foods Used in Low‐Income Countries and Implications for Bioavailability.” Food and Nutrition Bulletin 31, no. 2: S134–S146. 10.1177/15648265100312s206. 20715598

[fsn370264-bib-0024] Glinz, D. , R. Wegmuller , M. Ouattara , et al. 2017. “Iron‐Fortified Complementary Foods Containing a Mixture of Sodium Iron EDTA With Either Ferrous Fumarate or Ferric Pyrophosphate Reduce Iron Deficiency Anemia in 12‐ to 36‐Month‐Old Children in a Malaria Endemic Setting: A Secondary Analysis of a Cluster‐Randomized Controlled Trial.” Nutrients 9, no. 7: 759. 10.3390/nu9070759. 28708072 PMC5537873

[fsn370264-bib-0061] Gupta, A. K. , S. Samsher , and S. K. Goyal . 2006. “A Review*–*Jaggery A Healthy Sweetener.” International Journal of Agriculture Science 2, no. 1: 278–282.

[fsn370264-bib-0065] Hasan, M. N. , M. Z. Sultan , and M. Mar‐E‐Um . 2014. “Significance of Fermented Food in Nutrition and Food Science.” Journal of Scientific Research 6, no. 2: 373–386. 10.3329/jsr.v6i2.16530.

[fsn370264-bib-0025] Jain, P. , and M. Singh . 2021. “Production and Properties of Spray‐Dried Carrot Powder.” Pharma Innovation Journal 10, no. 2: 262–267.

[fsn370264-bib-0027] Karklina, D. , I. Gedrovica , M. Reca , and M. Kronberga . 2012. “Production of Biscuits With Higher Nutritional Value.” Proceedings of the Latvian Academy of Sciences, Section B. Natural, Exact, and Applied Sciences 66, no. 3: 113–116. 10.2478/v10046-012-0005-0.

[fsn370264-bib-0028] Khan, A. , and C. S. Saini . 2016. “Effect of Roasting on Physicochemical and Functional Properties of Flaxseed Flour.” Cogent Engineering 3, no. 1: 1145566. 10.1080/23311916.2016.1145566.

[fsn370264-bib-0029] Kumari, S. , V. Krishnan , M. Jolly , and A. Sachdev . 2014. “In Vivo Bioavailability of Essential Minerals and Phytase Activity During Soaking and Germination in Soybean (*Glycine max* L.).” Australian Journal of Crop Science 8, no. 8: 1168–1174.

[fsn370264-bib-0030] Lagnika, C. , P. A. F. Houssou , V. Dansou , et al. 2019. “Physico‐Functional and Sensory Properties of Flour and Bread Made From Composite Wheat‐Cassava.” Pakistan Journal of Nutrition 18: 538–547. 10.3923/pjn.2019.538.547.

[fsn370264-bib-0031] Maillet, J. , S. O'Sullivan , J. Skates , and E. Pritchett . 2005. “American Dietetic Association: Scope of Dietetics Practice Framework.” Journal of the American Dietetic Association 105: 634–640. 10.1016/j.jada.2005.02.001. 15800568

[fsn370264-bib-0032] Manary, M. J. , M. J. Ndkeha , P. Ashorn , K. Maleta , and A. Briend . 2004. “Home‐Based Therapy for Severe Malnutrition With Ready‐To‐Use Food.” Archives of Disease in Childhood 89, no. 6: 557–561. 10.1136/adc.2003.034306. 15155403 PMC1719944

[fsn370264-bib-0056] Nawaz, H. , M. A. Shad , R. Mehmood , T. Rehman , and H. Munir . 2015. “Comparative Evaluation of Functional Properties of Some Commonly Used Cereal and Legume Flours and Their Blends.” International Journal of Food and Allied Sciences 1, no. 2: 67–73. 10.21620/ijfaas.v1i2.12.

[fsn370264-bib-0033] Obinna‐Echem, P. , L. Barber , and C. Enyi . 2018. “Proximate Composition and Sensory Properties of Complementary Food Formulated From Malted Pre‐Gelatinized Maize, Soybean and Carrot Flours.” Journal of Food Research 7, no. 2: 17–24. 10.5539/jfr.v7n2p17.

[fsn370264-bib-0034] Olaitan, N. I. , M. O. Eke , and E. M. Uja . 2014. “Quality Evaluation of Complementary Food Formulated From *Moringa Oleifera* Leaf Powder and Pearl Millet (*Pennisetum Glaucum*) Flour.” International Journal of Engineering and Sciences 3: 59–63.

[fsn370264-bib-0036] Omolara, R. O. , and N. E. Victor . 2017. “Physicochemical and Rheological Properties of Complementary Diet From Blends of Maize, African Yam and Pigeon Pea Flour.” Scientific Journal of Food Science and Nutrition 3: 5–11.

[fsn370264-bib-0037] Onwuchekwa, A. I. , C. U. Charles , E. E. Ernest , O. A. Ahunna , S. I. Nwaorgu , and C. C. Aniemena . 2025. “Evaluation of Nutritional Qualities of Complementary Food Produce From Malted Rice, Soybean and Pumpkin Pulp Flour.” Food Chemistry Advances 6: e100863. 10.1016/j.focha.2024.100863.

[fsn370264-bib-0038] Papastylianou, P. , T. Ilias , R. Ioannis , and B. Dimitrio . 2019. “Sensitivity of Seed Germination to Salt Stress in Teff (*Eragrostis Tef* (Zucc.) Trotter).” Bulletin UASVM Horticulture 76: 91–95. 10.15835/buasvmcn-hort:2019.0004.

[fsn370264-bib-0039] Pasricha, S. R. , H. Drakesmith , J. Black , D. Hipgrave , and B. A. Biggs . 2013. “Control of Iron Deficiency Anemia in Low‐ and Middle‐Income Countries.” Blood 121, no. 14: 2607–2617. 10.1182/blood-2012-09-453522. 23355536

[fsn370264-bib-0040] Praveen Kumar, T. , R. K. Sahu , K. K. Sandey , and T. Rohit Kumar . 2017. “Importance of Oats in Human Diet: A Review.” Bulletin of Environment, Pharmacology and Life Sciences 7, no. 1: 125–130.

[fsn370264-bib-0057] Rashida Parvin, R. P. , M. A. Satter , S. A. Jabin , et al. 2014. “Studies on the Development and Evaluation of Cereal Based Highly Nutritive Supplementary Food for Young Children.” International Journal of Innovation and Applied Studies 9, no. 2: 974–984.

[fsn370264-bib-0058] Robin, J. M. 2017. “Mineral Nutrient Composition of Vegetables, Fruits and Grains: The Context of Reports of Apparent Historical Declines.” Journal of Food Composition and Analysis 56: 93–103. 10.1016/j.jfca.2016.11.012.

[fsn370264-bib-0041] Rohner, F. , M. B. Zimmermann , R. J. Amon , et al. 2010. “In a Randomized Controlled Trial of Iron Fortification, Anthelmintic Treatment, and Intermittent Preventive Treatment of Malaria for Anemia Control in Ivorian Children, Only Anthelmintic Treatment Shows Modest Benefit.” Journal of Nutrition 140, no. 3: 635–641. 10.3945/jn.109.114256. 20107144

[fsn370264-bib-0042] Roshana, M. R. , and T. Mahendran . 2019. “Nutritional and Sensory Evaluation of Carrot Flour‐Incorporated Complementary Food Mixtures for Infants.” Sri Lanka Journal of Food and Agriculture 5, no. 2: 27–32. 10.4038/sljfa.v5i2.74.

[fsn370264-bib-0043] Roy, D. 2018. Development of Carrot Powder Added Mozzarella Cheeses. Project Report Submitted to Daffodil International University.

[fsn370264-bib-0044] Scherz, H. , and G. Bonn . 2008. Analytical Chemistry of Carbohydrates. Georg Thieme Verlag.

[fsn370264-bib-0045] Shad, M. S. , H. Nawaz , M. Hussain , and B. Yousuf . 2011. “Proximate Composition and Functional Properties of Lotus Rhizomes (*Nelumbo Nucifera*) From Punjab, Pakistan.” Pakistan Journal of Botany 43, no. 2: 895–904.

[fsn370264-bib-0046] Shahid, M. , S. B. Masood , M. A. Faqir , and N. Haq . 2008. “Baking and Storage Stability of Retinyl Acetate (Vitamin A) Fortified Cookies.” Pakistan Journal of Nutrition 7, no. 4: 586–589. 10.3923/pjn.2008.586.589.

[fsn370264-bib-0047] Sirό, I. E. , B. Apolna , and A. Lugasi . 2008. “Functional Food: Product Development, Marketing and Consumer Acceptance—A Review.” Appetite 51, no. 3: 456–467. 10.1016/j.appet.2008.05.060. 18582508

[fsn370264-bib-0048] Tabitha, M. A. , G. A. Issac , and A. A. Christiana . 2020. “Formulation, Proximate Analysis, and Sensory Evaluation of Mumu From Pearl Millet, Irish Potato, and Sesame Seed Blend.” Agricultural Sciences 11, no. 3: 235–246. 10.4236/as.2020.113015.

[fsn370264-bib-0049] Tang, G. , J. Qin , G. G. Dolnikowski , R. M. Russell , and M. A. Grusak . 2005. “Spinach or Carrots Can Supply Significant Amounts of Vitamin A as Assessed by Feeding With Intrinsically Deuterated Vegetables.” American Journal of Clinical Nutrition 82, no. 4: 821–828. 10.1093/ajcn/82.4.821. 16210712

[fsn370264-bib-0062] Tesfaye, A. , G. Feyera , W. Aselefech , and E. Tarekegn . 2020. “Chemical Formualtion and Characterization of Complementary Foods From Blend of Orange‐Fleshed Sweet Potato, Brown Teff, and Dark Red Kidney Beans.” International Journal of Food Science 2020: e4803839. 10.1155/2020/4803839. PMC7244972 32509844

[fsn370264-bib-0050] UICEC , and WHO . 2005. Global Action Against Cancer Now. UICC and WHO Publications Departments.

[fsn370264-bib-0051] USDA . 2009. National Nutrient Database for Standard Reference. US Department of Agriculture.

[fsn370264-bib-0064] Kuchtová, V. , Z. Kohajdová , J. Karovicova , and M. Lauková . 2018. “Physical, Textural, and Sensory Properties of Cookies Incorporated With Grape Skin and Seed Preparations.” Polish Journal of Food Nutrition and Science 68, no. 4: 309–317. 10.2478/pjfns-2018-0004.

[fsn370264-bib-0059] Wack, R. P. , E. L. Lien , D. Taft , and J. D. Roscelli . 1997. “Electrolyte Composition of Human Breast Milk Beyond the Early Postpartum Period.” Nutrition 13, no. 9: 774–777. 10.1016/S0899-9007(97)00187-1. 9290089

[fsn370264-bib-0052] Wakil, S. M. , and J. O. Ola . 2018. “Development of Maize‐Tigernut Fortified Weaning Food Using Starter Cultures.” Food and Nutrition Sciences 9: 1444–1457. 10.4236/fns.2018.912105.

[fsn370264-bib-0053] WHO/FAO . 2016. “Library Cataloguing‐in‐Publication Data.” In Nutritional Requirements in Human Nutrition, 10th ed. WHO/FAO.

[fsn370264-bib-0054] Yoon, J.‐W. , S.‐I. Ahn , H.‐N. Kim , et al. 2017. “Qualitative Characteristics and Determining Shelf‐Life of Milk Beverage Products Supplemented With Coffee Extracts.” Journal of Food Science and Animal Resources 37, no. 2: 305–312. 10.5851/kosfa.2017.37.2.305. PMC5434217 28515654

[fsn370264-bib-0055] Zhang, G. , Z. Xu , Y. Gao , X. Huang , and Y. Zou . 2015. “Effects of Germination on the Nutritional Properties, Phenolic Profiles, and Antioxidant Activities of Buckwheat.” Journal of Food Science 80, no. 5: H1111–H1119. 10.1111/1750-3841.12830. 25858540

